# Identification of New Molecular Entities (NMEs) as Potential Leads against Tuberculosis from Open Source Compound Repository

**DOI:** 10.1371/journal.pone.0144018

**Published:** 2015-12-07

**Authors:** Sudha Sravanti Kotapalli, Sri Satya Anila Nallam, Lavanya Nadella, Tanmay Banerjee, Haridas B. Rode, Prathama S. Mainkar, Ramesh Ummanni

**Affiliations:** 1 Centre for Chemical Biology, CSIR-Indian Institute of Chemical Technology, Hyderabad - 500 007, India; 2 Division of Natural Product Chemistry, CSIR-Indian Institute of Chemical Technology, Hyderabad - 500 007, India; 3 Syngene International Ltd., Biocon park, Bangalore - 560 099, India; University of Padova, Medical School, ITALY

## Abstract

The purpose of this study was to provide a number of diverse and promising early-lead compounds that will feed into the drug discovery pipeline for developing new antitubercular agents. The results from the phenotypic screening of the open-source compound library against *Mycobacterium smegmatis* and *Mycobacterium bovis* (BCG) with hit validation against *M*. *tuberculosis* (*H37Rv*) have identified novel potent hit compounds. To determine their druglikeness, a systematic analysis of physicochemical properties of the hit compounds has been performed using cheminformatics tools. The hit molecules were analysed by clustering based on their chemical finger prints and structural similarity determining their chemical diversity. The hit compound library is also filtered for druglikeness based on the physicochemical descriptors following Lipinski filters. The robust filtration of hits followed by secondary screening against BCG, *H37Rv* and cytotoxicity evaluation has identified 12 compounds with potential against *H37Rv* (MIC range 0.4 to 12.5 μM). Furthermore in cytotoxicity assays, 12 compounds displayed low cytotoxicity against liver and lung cells providing high therapeutic index > 50. To avoid any variations in activity due to the route of chemical synthesis, the hit compounds were re synthesized independently and confirmed for their potential against *H37Rv*. Taken together, the hits reported here provides copious potential starting points for generation of new leads eventually adds to drug discovery pipeline against tuberculosis.

## Introduction

Worldwide, tuberculosis accounts for more deaths among people than all other infectious diseases combined [1]. Tuberculosis is a highly infectious disease caused by *Mycobacterium tuberculosis (M*. *tb)*. Its target organ is mostly lungs, especially alveolar macrophages. It has been recorded that tuberculosis is the second largest infectious disease responsible for mortality in the world [2]. Though different regimens are available for tuberculosis (TB) treatment, more than two million people die from TB every year [3]. However, effective treatment of TB is challenging to date. First-line treatment with standard drugs such as rifampicin, isoniazid, ethambutol and pyrazinamide for a period 6–8 months is associated with non-compliance among TB patients, which leads to evolution of the multi- (MDR) and extensively drug-resistant (XDR) strains. Among the XDR patients mortality rate is as high as 100% [4,5]. Some of the newly emerged strains of the bacteria do not respond to drugs of first line treatment such as isoniazid and rifampicin, the two most powerful (or standard) anti TB drugs. In some countries MDR/XDR strains can account for up to 22% of infection [6]. These drug resistant strains pose a greater threat to the health of the people due to no efficacious treatments till date [5]. The available line of treatment for tuberculosis is completely ineffective against these strains. Another major challenge is to combat bacteria from the latently infected individuals who stand the risk of progressing to active TB. Only within the last few years a few promising drug candidates have emerged [7–9]. Therefore, there is an urgent need to develop new drugs to decrease the time span of TB treatment including drug resistant and latent TB infections; while also reducing the occurrence of any of the possible side effects known for existing drugs.

In the recent past, combinatorial chemistry emerged as a key platform to develop diverse chemicals for drug discovery. However, it is necessary to explore the chemical space of the compounds for various biological applications using different screening methods. Though random screening of compounds is less efficient, it has led to the identification of new scaffolds as potential leads. The target based or whole cell based screening methods are general approaches used in early drug discovery. An advantage of target based approach is that the selected target being present only in bacteria not in humans will reduce the toxicity problems in further stages of drug development. However, these compounds identified in target based approach needs to reach their target in the bacteria which is often a bottleneck due to the unique and complex cell wall of *M*. *tb*. In addition, bacteria are equipped with efflux pumps that remove drugs from the cells lowering the effective concentration of these drugs against the target.

Therefore, for discovering new antitubercular agents using “whole-cell” based approach gained better appeal than target based approach. In this whole-cell based approach, compounds are chosen based on their ability to kill the bacteria. Compounds identified in whole-cell screening already fulfil some important criteria such as, cell permeability of drugs and potent inhibition against *Mycobacterium*. The concerns about possible toxicity are also parallelly followed by evaluating the lead compounds in battery of assays related to safety profiling. Further elucidation of the compound’s mechanism of action can aid a step forward to develop new antitubercular agents. Thus, these compounds provide suitable chemical and biological starting points to further develop drug like scaffolds with the help of medicinal chemistry principles. Therefore, in the current study we have explored a library of compounds (both natural product analogs and diverse new chemical entities) archived at the National Mol Bank facility to identify novel hits with antitubercular properties. The hits identified have been tested for cytotoxic potential and analyzed for physicochemical properties. Till date many compound libraries are been screened for the identification of starting points for various drug discovery programmes [10–12]. The poor success rate in antibacterial drug discovery is mainly attributed to the lack of chemically diverse compounds. The compound library screened against *Mycobacterium* in the present study is comprised of chemically diverse compounds containing compound classes like, flavonoids, carbohydrates, various heterocycles, steroids, peptides and most importantly the compounds of natural origin with chemically diverse scaffolds.

## Materials and Methods

### Microbial strains and Cell lines

The bacterial strains used in the current study were obtained from authenticated sources (ATCC, USA). The lyophillized strains were revived in Middlebrook 7H9 broth supplemented with Glycerol, 0.05% v/v Tween 80 and 10% ADS (Albumin-Dextrose-Saline) (Sigma) for *Mycobacterium smegmatis* (mc^2^155)and 0.05% Tween 80 (Sigma) and 10% OADC (0.06% oleic acid, 5% BSA, 2% Dextrose, 0.85% NaCl), glycerol (0.2% v/v) (Himedia) for *Mycobacterium bovis* (BCG) and *M*. *tb (H37Rv)*. Morphology of the revived bacteria is verified by streaking small loopfull of cultures on Middlebrook 7H10 agar along with required supplements. The cultures were stained with standard Ziehl-Neelsen stain on regular basis to confirm *Mycobacterium*. All bacterial strains were grown at 37°C with shaking at 200 rpm.

The cell lines HepG2 (human hepatoma cells) and A549 (human lung epithelial cells) used in the current study were obtained from ATCC (Manassas, USA) and cultivated in DMEM (Sigma) supplemented with 10% foetal bovine serum, 100 units/mL penicillin and streptomycin. Cells were regularly tested for mycoplasma contamination using the MycoAlert Kit (Cambrex Bio Science Rock- land, Inc., Rockland, ME, USA).

### Chemical libraries for screening for antimycobacterial compounds

CSIR-Indian Institute of Chemical Technology (IICT) as a Council for scientific and industrial research (CSIR) laboratory is provided with National Mol Bank (NMB) for storing New Molecular Entities (NMEs). The facility has stored synthetic/isolated scaffolds from different research groups and commercial sources for identifying new chemical moieties for various diseases. Currently the facility houses more than 10000 compounds with vast chemical diversity from different sources and stored in bar coded vials (50mM in DMSO) in 96 well plate formats. The daughter plates (10 mM) were prepared from stocks for screening in different bio assays. The scaffolds are chosen based on their chemical categorisation and source. Natural products isolated get preference over commercially available common scaffolds. The choice of natural product like skeleton has been the main criterion even in commercial libraries. The classes of compounds range from flavonoids, terpenoids, carbohydrate based, peptides and peptido-mimetic, heterocyclic compounds, lipids and alkaloids and derivatives thereof. As the primary screening involves a large number of compounds, all compounds were screened at a fixed single concentration of 30μM. The choice of the concentration is due to fact that the compounds require higher concentration to inhibit growth of *M*. *smegmatis* compared to *M*. *tuberculosis*.

### Antimycobacterial Assay

*M*. *smegmatis* and *M*. *bovis (BCG)* Pasteur are surrogate models for large scale screening of chemicals to identify new antimycobacterial agents [7,13–15]. The primary screening at single concentration (30 μM) was performed against *M*. *smegmatis* in 96-well flat-bottom polystyrene microtiter sterile plates (Nunc). For primary screening, the test compounds prepared in DMSO or DMSO alone as control were dispensed in to test plates (in designated triplicate wells) prior to addition of the assay components. Using a hand pipette, a 98 μl of inoculum (over night culture with 0.6 OD diluted at 1:1000 in 7H9 broth) was distributed into sterile micro titre plates. This dilution of test compounds with inoculums gives 30 μM of final concentration of the compounds in screening medium. To better ascertain the activity of the compounds, controls like DMSO as a solvent control, media control (Blank) as well as Rifampicin and Isoniazid were added as positive controls for inhibition of *M*. *smegmatis* growth in every plate. The peripheral wells of assay plates were filled with sterile distilled water to avoid evaporation in assay wells. Inoculated plates were stacked in groups of 7–8 plates. Plates were carefully wrapped with aluminium foil to prevent evaporation and allowed to incubate at 37°C at 80% relative humidity. The incubation time was for four days in case of BCG and 32 hours for *M*. *smegmatis*. After the incubation period, the growth of the bacteria was studied by turbidometry measuring the absorbance at 600 nm using a Multi Mode Reader (Perkin Elmer). Percentage growth inhibition was determined against DMSO control (the compound dilutions were prepared in DMSO). The growth inhibition effect of compounds was calculated as:

 % Inhibition = 100× [OD with compound –OD of Negative control]  % [OD of Positive control –OD of Negative control]

Those compounds which showed 40% of inhibition or more were further analyzed to determine their MIC values against *M*. *bovis* and *M*. *smegmatis*. From the results obtained in primary screening, compounds showing more than 40% growth inhibition were reconfirmed in same analogy with freshly prepared stocks from original compounds preserved up on submission of samples to NMB. To determine MIC value of test compounds, a full dose response was performed by taking 0 to 100 μM of final concentration of compounds to cells in assay plates. After the period of incubation, from the absorbance of the inoculum observed at 600 nm, MIC values were calculated as the lowest drug concentration, which showed 90% growth inhibition of the bacteria. In every assay plate negative control DMSO was added in one column which corresponds to 100% growth, and positive controls (Rifampicin and Isoniazid) were added in one column. These controls were used to monitor assay quality as well as for normalizing the data on a per-plate basis.

### M. tuberculosis (H37Rv) inhibition assay

To determine efficiency of the hits identified from primary screening (compounds inhibiting growth of *M*. *smegmatis* and *M*. *bovis*) selected compounds have been tested against *M*. *tuberculosis*. The measurement of the minimum inhibitory concentration (MIC) for each tested compound was performed in 96 well flat-bottom polystyrene microtiter sterile plates. From the 10 mM stock solutions, nine of two fold drug dilutions in neat DMSO starting at 10 mM were performed (lines B–F, rows 2–10 of the plates, plate format is retained between master and daughter plates). Nine of two fold dilutions (64 to 0.25 μg/mL) of Isoniazid as positive control were prepared (lines G, rows 2–10). These compound solutions (4 μl) were added to daughter plates in duplicates. Neat DMSO (4 μL) was added to row 11 (growth and blank controls). The inoculum was standardized to ~1×10 CFU/mL and diluted 1:1000 in Middlebrook 7H9 broth with required supplements to produce final *H37Rv* inoculum. This inoculum (200 μL) was added to all plates loaded with compounds and controls entire plate except A and H-rows as well as 1st column (blank controls filled with H_2_O). Inoculated plates were sealed with parafilm and stacked in groups of 7–8 plates. Plates were carefully wrapped with aluminium foil to prevent evaporation and allowed to incubate at 37°C without shaking for six days. After incubation period, 5 μL of freshly prepared Alamar Blue reagent in sterile phosphate-buffered saline (PBS) was added in each well. The assay plates were further incubated for 24 h at 37°C. In Alamar Blue Assay, blue colour in wells stands for no growth while appearance of pink colour showed growth of bacteria. MIC was determined by comparison of growth in compound wells with control in column 11 within the every assay plate.

### Cytotoxicity assays

The cytotoxicity of the compound was tested by performing a Sulforhodamine B Assay (SRB) using A549 and HepG2 cell lines. For cell proliferation assays, the cell line of interest was seeded in flat bottom 96-well plate (5000 cells/100 μL) in a medium containing 10% serum. The plates were incubated for 18–20 h in an incubator with continuous supply of 5% CO_2_ to ensure proper adherence of the cells to the surface bottom of the wells. After 18 h the cells were treated with the compound. The compounds were prepared at 50 fold higher concentrations to obtain required final concentration to cells. From source plates, 2 μl aliquot was added to the each well, thereby making the final concentration of compound 0 to 100 μM. Each compound was tested in triplicate and the cytotoxicity was determined as the average of that triplicate. DMSO and Doxorubicin (as standard anticancer drug) were taken as vehicle and positive controls respectively. Further, the plates were incubated for another 48 h in an incubator maintained at 37°c with a constant supply of 5% CO_2_. After 48 h, cells were fixed using 10% TCA solution and incubated for 1 h at 4°C. Then plate was rinsed carefully with MQ water and air dried at room temperature. After adding 0.057% SRB solution plate was incubated for approximately 30 min before it was rinsed off using 1% acetic acid. The plates were then air dried and 100μL of 10mM Tris base was added to each well to solubilise the SRB to measure the absorbance using Perkin –Elmer Multimode Reader at 510nm. The measure of absorbance is directly proportional to cell growth and is used to calculate the IC_50_ values. Further, therapeutic index (TI) values are calculated for the test compounds. TI is the ratio of concentration of compound which causes 50% toxicity to the cells divided by concentration which causes 50% effective growth inhibition of the target organism. Thus, the therapeutic index was calculated using the formula IC_50_/GI_50_ (concentration which caused 50% growth inhibition of *Mycobacterium tuberculosis* (*H37R*_*v*_) [16,17].

### Computational Analysis

As mentioned in above section, results from primary screening of compounds library against results *M*. *smegmatis* were collected as percentage growth inhibition by compounds compared to control DMSO (100% growth). In primary screening each compound was screened in duplicate, and composite z-scores were calculated using percentage growth inhibition and standard deviation values using DMSO and Rifampicin controls as references. The Z score was calculated as

 z score = % Inhibition – Average Mean ÷ Standard deviation

The cut-off mean z-score of the data is set to select compounds showing 50% inhibition in primary assays. Hits from the *M*. *smegmatis* screen were defined as compounds with a composite z-score of more than 1.5. Further to determine drugability of hit chemicals identified in primary screen a systematic analysis has been performed using cheminformatics tools. In current study we have determined physicochemical properties such as solubility, permeability and Lipinski’s rule including molecular weight, number of hydrogen bond acceptors and donors, logP and polar surface area (PSA) using Cytoscape and ChemMine tools available as open source. Based on the calculated parameters of compounds the identified hit list has been filtered to exclude compounds that do not satisfying the criteria within pre-established drug-like parameters [11].

Hierarchical Clustering of primary hits has been performed to understand their uniqueness and pattern analysis to identify signature structure showing good antimycobacterial activity. Structurally similar molecules clustering together usually have similar physicochemical and biological properties. We have clustered molecules using pubchem fingerprints as implemented in the chemistry development kit (CDK) [18]. To cluster molecules, we have developed two scripts TD_calculator and Taylor_Butina written in PERL 1.0. TD_calculator was developed to read pubchem fingerprints output from CDK descriptor calculator (v1.3.2). TD_calculator was used to look into similarity of the each molecule within set of molecules to be clustered. Similarity between molecules was measured using Tanimoto distance. We tried different Tanimoto distances to define similarity between two molecules using molecules from molecule bank. A cut-off of 0.15 gave us chemically meaningful clusters. Metadata containing similarity information of each molecule found against other molecules were used as input to our next script Taylor_butina. This implementation is similar to one described by Butinaet. al. [19]. Molecules are ordered in a descending fashion, according to number of neighbours. Molecule with largest number of neighbours forms the biggest cluster. In case a molecule appears in more than one cluster, then it is attached to the cluster which has larger number of molecules. Clustered compound families were represented using a visualization tool Cytoscape 3.1. The 2D images of the compounds are displayed by providing the InCHI or SMILES strings in chemViz.

### Minimum Bactericidal Concentration (MBC) Test

MBC test determines the minimum concentration of antimicrobial agent required to prevent the growth of an organism after sub-culture on to an antibiotic-free media. The MBC test allows determining whether the antimicrobial agents show bactericidal or bacteriostatic effect on growth of particular microorganism [20]. Thus, as we have already determined the MIC values for the hit compounds identified, the same compounds were subjected to MBC determination. To perform MBC test, as reported earlier a conventional CFU approach was adapted for all test organisms in the present study with minor modifications [21]. A pure bacterial culture grown overnight diluted in complete growth medium to a concentration between 1 x 10^5^ and 1 x 10^6^ CFU/ml. A series of test compound dilutions (maximum 100 times of MIC observed for respective compounds) are prepared in 96 well microtiter plates. The bacteria were first exposed to the compounds by inoculating the bacteria with the respective compounds at varying concentrations including the MIC concentrations. All dilutions of the test compounds are inoculated with equal volumes of the specified microorganisms independently and cultured for further 24 h. Then, 100 μL of these inoculums from each dilution was then spread on an antibiotic free M7H10 solid agar medium supplemented with OADC and Glycerol. The plates were incubated for the duration of 7 days, during which the growth of *Mycobacterium* was observed on a daily basis. A positive control well is included for test microorganisms to demonstrate satisfactory microbial growth over the course of the incubation period and negative control to ensure media sterility for long incubation period. Upon the completion of the incubation period, the growth in the test plates was compared to control plates spread with inoculums treated with DMSO. The differences in occurrence of colony formation units (CFU) in test as well as controls were used to measure the MBC of the compounds. The MBC is the lowest concentration that demonstrates a pre-determined reduction (such as 99.9%) in CFU/ml when compared to the MIC dilution. Based on the determined MIC and MBC values for a respective compound, it can be labelled as either bactericidal or bacteriostatic against a particular microorganism.

## Results and Discussion

### *M*. *smegmatis* primary screening

To evaluate compound collection at CSIR-IICT’s National Mol Bank (10,000unique chemical entities) against mycobacteria while avoiding large culture volumes of infective material within a bio safety level (BSL) 3 environment, we decided to use *M*. *smegmatis (MC* ^*2*^ *155)* as an *M*.*tuberculosis* surrogate for early drug screening purpose. *M*. *smegmatis* is an aerobic, fast growing, non-pathogenic mycobacterium and can be worked safely within a BSL1 environment. *M*. *smegmatis* has many common features with pathogenic mycobacteria *H37Rv* [22]. This species shares >2000 gene homologs with *M*. *tuberculosis* and it has same unusual cell wall structure like *M*. *tuberculosis* and other mycobacterial species [23]. There are several reports on the use of *Mycobacterium smegmatis* as a primary screen to select compounds which could be active against *M*. *tuberculosis* [7,24,25]. It should be noted that the new antitubercular drug diarylquinoline known as TMC207 (Bedaquiline) approved in 2013 was identified from a high throughput screening using *M*. *smegmatis* [7]. Therefore, we believed that screening rare library of small molecules archived in our facility will identify potential compounds interesting to develop new antitubercular agents.

**Fig 1:**
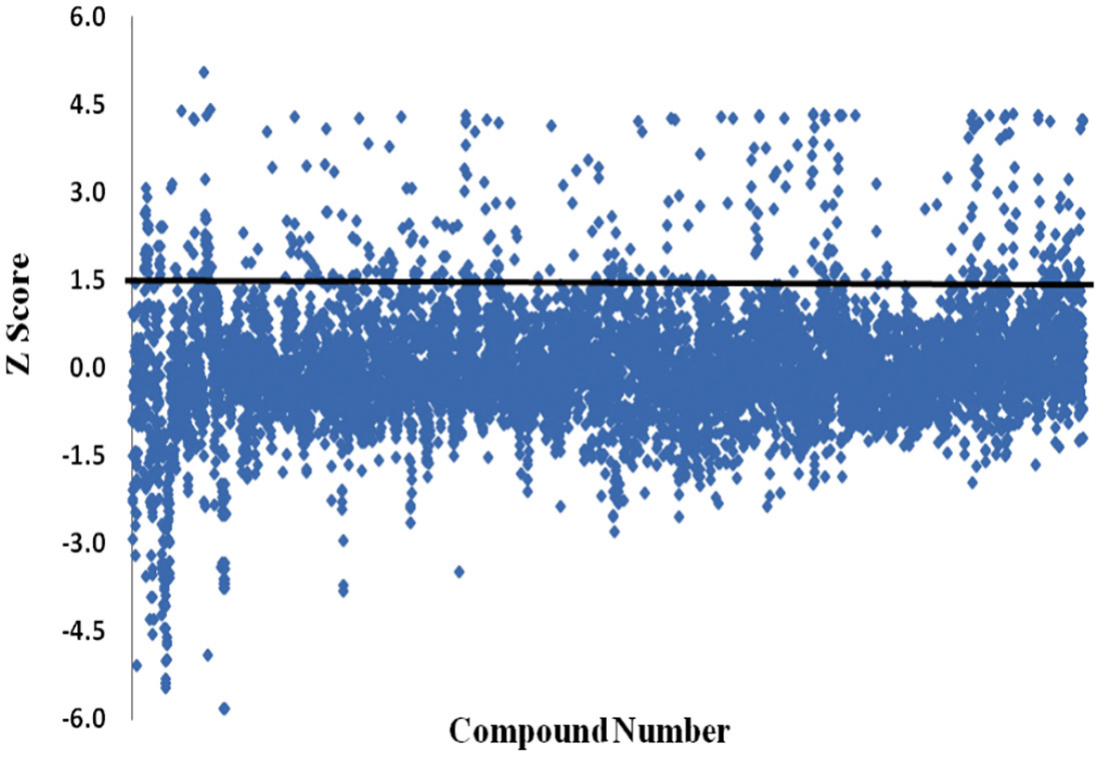
Primary screening results. Using a z-score cutoff of 1.5, 150 compounds were identified actives against mycobacteria. The distribution of the z-score for the primary screen is shown here.

**Fig 2:**
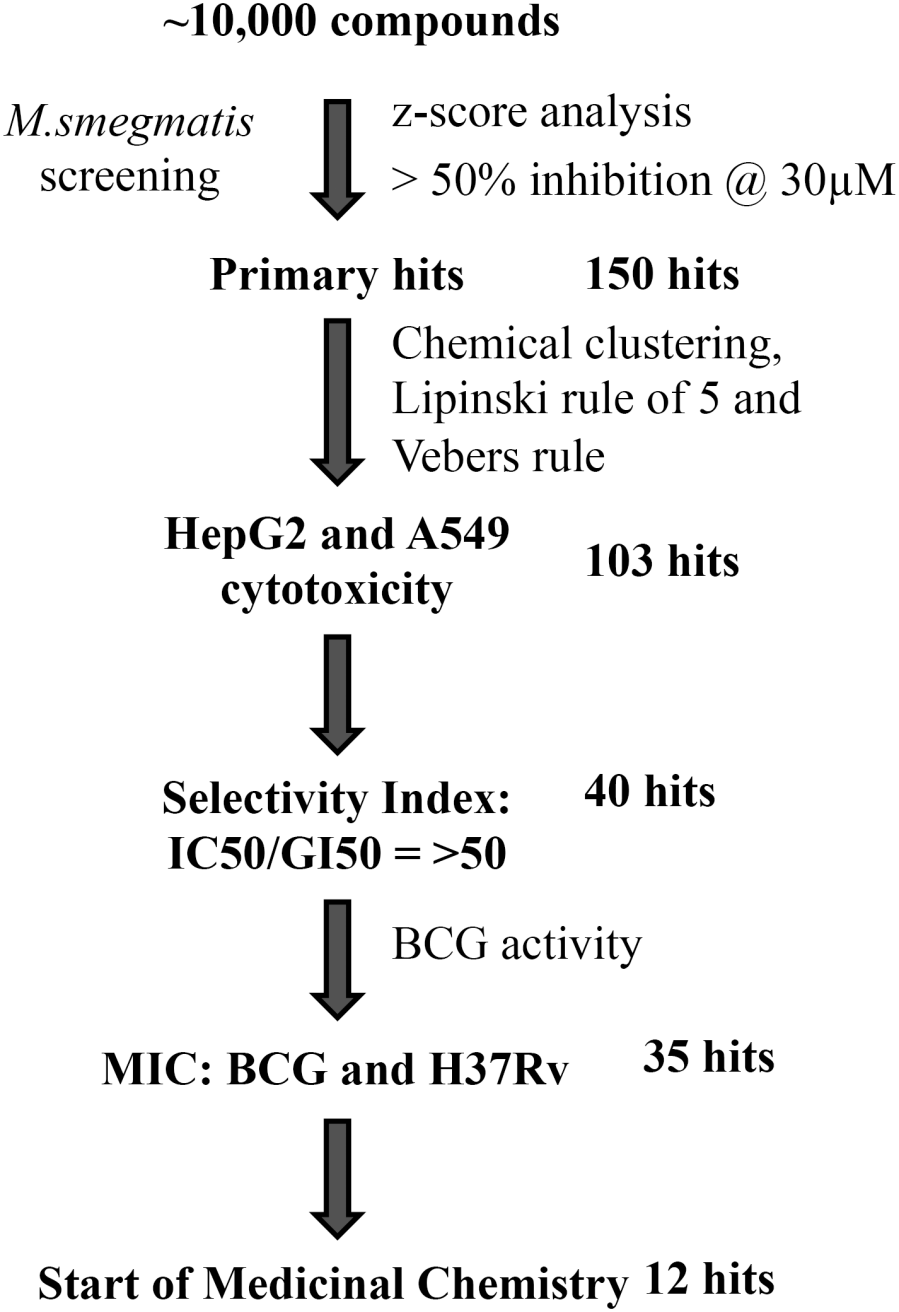
Work flow. Small molecule library screening for anti mycobacterial potential, physicochemical properties following medicinal chemistry principles and cytotoxicity identified potential hit scaffolds.

*M*.*smegmatis* was grown under identical culture conditions for different batch of screening assays and full library of compounds screened in triplicates. The z-score was calculated to determine the reliability of the screens. The mean z-score from screening results was calculated to determine compounds inhibiting bacterial growth. The mean z-score of the primary screening data is 7.34 and standard deviation is 21.47. The mean z-score for Rifampicin and Isoniazid were 4.4 and 4.9 respectively. After obtaining the standard deviation and average mean, the z score was calculated for the entire data set. A minimum cut-off of composite z-score of more than 1.5 (minimum 50% growth inhibition) of the library compounds at 30 μM was applied to describe compounds as antimycobacterial [12]. Using these criteria, 150 hits were detected against *M*. *smegmatis* from primary screening (Fig 1). The initial *M*. *smegmatis* primary hit list was narrowed after application of number of stringent filters following favourable guidelines selecting chemicals with drug likeness. The primary screening and hit progression cascade is presented in Fig 2.

### Screening of primary hits for drug like Parameters

**Fig 3:**
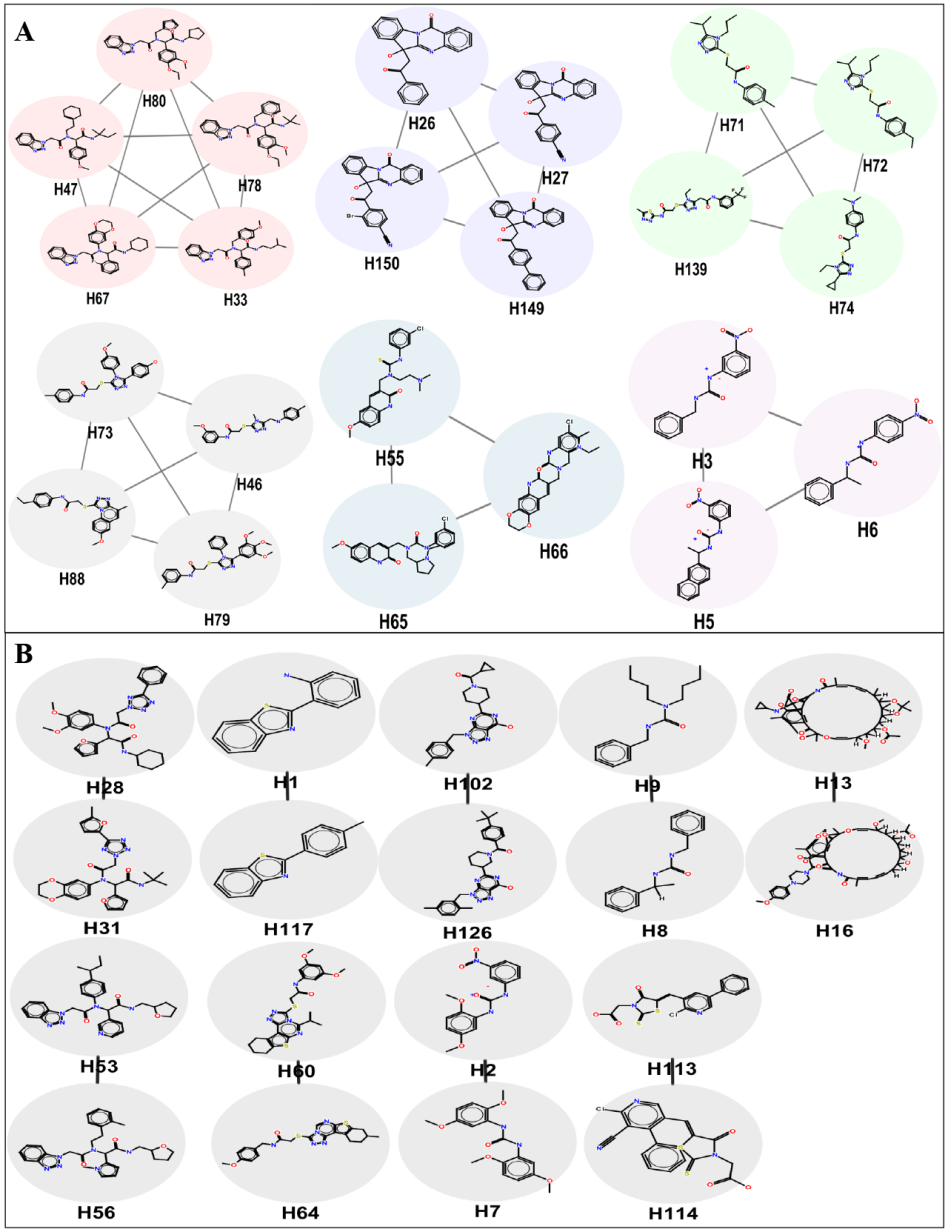
Clustering of actives identified in primary screening. All hit compounds identified were chemical clustered using pubchem finger prints. (A) The major cluster with five compounds maximum to minor cluster with three compounds and (B) doubles tons are shown here.

Analysing small molecule screening data is an important component of drug discovery pipeline [26–28]. Cheminformatics tools enable exploration of the structural similarities, physicochemical properties and bioavailability of the hits from primary screening before proceeding further in drug discovery research. Clustering of small molecules based on the structural similarity scores has been performed. The hits obtained in primary screening resulted in the formation of 6 major cluster families, 9 doubletons (Fig 3A and 3B) and remaining 109 singletons (i.e. unclustered compounds which remain single). There are several approaches for clustering of small molecules. Among commonly used methods, the structure based approaches using classical descriptors such as chemical finger prints have been widely used. These clustering parameters will help in scaffold hopping to compare and prioritize structurally related new lead molecules from random screening approaches. From the observed clustering pattern it is clear that only minor portion of hits (23 molecules) have structural similarity where as 109 compounds displayed as unique scaffolds (Fig 3A and 3B). As clustering of compounds based on structure is the best approach to determine structural redundancies and diversity analysis, our analysis clearly shows that the primary hits are heterogeneous. Therefore, not to lose any good scaffolds in discovery pipe line, we have considered all these compounds to perform further screening to identify potential molecules active against pathogenic bacteria with minimal toxicity in cellular models.

In the drug discovery pipeline, many of the newer drugs like molecules (hits /leads) often fail in advanced discovery phase. Therefore, it is important to assess ‘druglikeness’ and ‘leadlikeness’ of potential hits obtained from comprehensive screening approach. They are also useful for enriching library collection with advantageous properties for optimization of hits to obtain leads /new drugs [29,30]. Lipinski's rule of five is a rule of thumb to determine drug likeness of a chemical compound with properties that would likely make it orally active drug. These parameters were set based on the observation that most orally administered drugs are small and moderately lipophilic molecules [31]. In drug discovery chain, candidate drugs that authenticate these rules tend to have less attrition rates for failure in clinical trials and hence have an increased chance of reaching the market [31,32]. Therefore, to narrow the list of hits from primary screening we have applied several stringent filters for obtaining potential hits to go forward developing new antitubercular agents.

**Fig 4:**
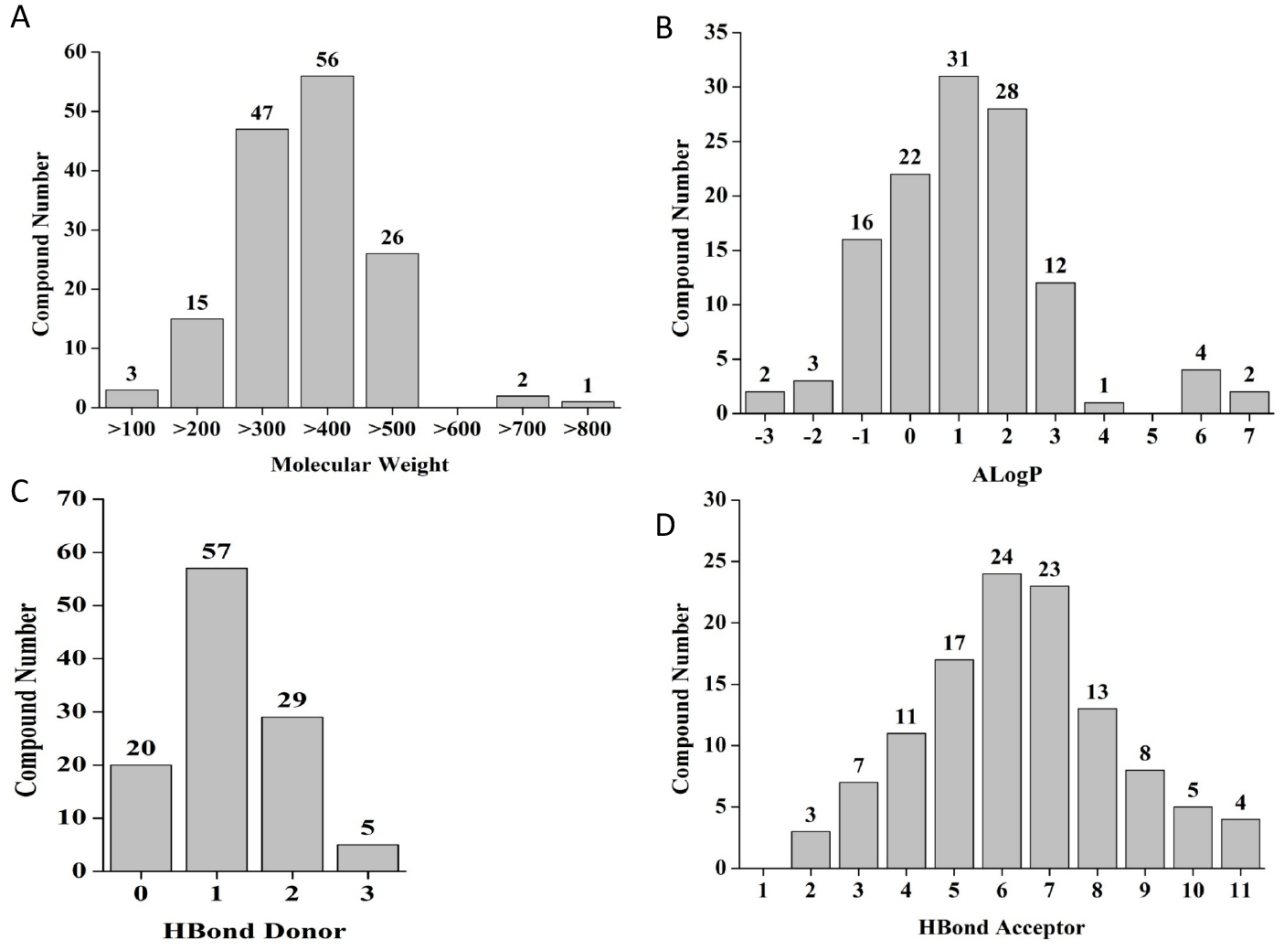
The hit compounds were analyzed by Cytoscape and Chemmine for physicochemical parameters. Filtering compounds based Lipinski rule of five druglikeness (A) Molwt cutoff, (B) alogP (C) Hydrogen bond donors and (D) Hydrogen bond acceptors.

As the first filter, Lipinski’s rule of five is applied to all hits obtained. The 150 compounds were filtered for allowed molecular weight (≥ 180 and ≤ 500 Da), aLogP (≤ 5), number of hydrogen bond donors (≤ 5) and acceptors (≤ 10). From the selected hits, 32 compounds did not meet the set criteria for molecular weight of ≥ 180 and ≤ 500 Da and they were excluded from auxiliary analysis performed (Fig 4A). Increase in Molecular weight of the compound may lead to decrease in solubility and subsequent bioavailability of the compound [33]. For remaining 121 compounds we have calculated aLogP using Cytoscape [34]. It is a measure of solubility using partition coefficient which determines solubility of the compound in two immiscible phases, i.e octanol and water. From the values obtained it was clear that all 115 compounds except 6 fall in the range with logP ≤5 (Fig 4B) thereby making these compounds most likely soluble in aqueous solutions. The resultant 115 compounds were then subjected to a further analysis for calculating H bond donors (sum of N-H and O-H) and acceptors (sum of N and O atoms). An online application tool ChemMine was used to calculate the described parameters [35]. For drug likeness of any molecule the total number of hydrogen bond donors as well as acceptors is an imperative chemical feature. An increase in number of hydrogen bonds reduces partitioning from the aqueous phase into the lipid bilayer membrane affecting its permeability through cell membranes [29]. Therefore, it is important to keep minimum number of H bond donors in NME for their drug likeness. Thus, the cut off value for H bond donors was taken as ≤5 and a cut off value of ≤10 was taken for H bond acceptors. As is evident from the graphical representation (Fig 4C and 4D), 4 compounds did not follow the given criteria of cut off values. Hence, as suggested by Lipinski’s rule of 5, taken together 111 compounds met the stringent criteria to be considered for further validation.

**Fig 5:**
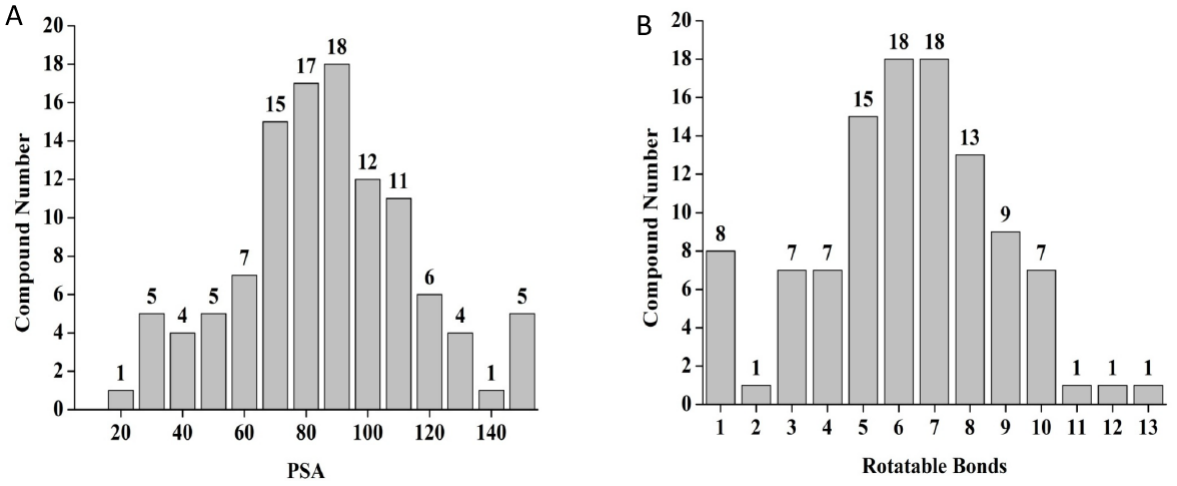
To improve the predictions of druglikeness other filters have been added to the analysis. The list of identified hit compounds have been filtered based on Veber Rules (A) PSA and (B) Number of rotatable Bonds.

To improve the predictions of drug likeness, the Lipinski’s rules were refined [36] including rotatable bonds and polar surface area (PSA). PSA is a sum of polar atoms which determines permeability of drugs. It has been reported that the PSA and the number of rotatable bonds have been found to be valuable parameters to discriminate orally active compounds from a large set of compounds [37]. In particular, compounds with ≤10 rotatable bonds and PSA ≤140 Å^2^ are predicted to have good bioavailability [37]. Lead drug molecules with a PSA cut off value ≤140Å^2^ tend to be more permeable through cell membranes. However, for molecules to be permeable through the blood–brain barrier PSA should be ≤90Å^2^ [38]. To exclude any chemicals which do not show good calculated permeability, PSA was calculated for short listed chemicals applying ChemMine. Based on the allowed PSA values and rotatable bonds, 8 compounds which did not show good calculated permeability properties were dismissed. Thus, a set of 103 compounds were selected for further bio assays (Fig 5).

### *Mycobacterium tuberculosis* (*H37Rv*) and cytotoxicity screening: the lead progression

The above selection criteria have identified 103 compounds with physiochemical properties of a drug like molecule. Thus, these compounds were subjected to cytotoxicity screening against selected cell line A549 (lung epithelial cancer cells) and HEPG2 (Hepatocellular liver carcinoma). From the cell viability results using both the cell types it is clear that 40 compounds showed selectivity index of > 50 towards antimycobacterial activity than cytotoxicity. As there is a debate on surrogate mycobacterium strains best suitable for screening new compound libraries, the 40 hits filtered from primary screening were screened against *M*.*bovis* (BCG) prior to test against *H37Rv*. It has been reported that BCG is a more sensitive model for screening of small molecule libraries which can be active against *M*. *tuberculosis* [7,13–15]. From the secondary screening 35 compounds showed potent MIC values of <25μM. It is evident that 78% compounds identified from *M*. *smegmatis* screen have showed activity against *M*. *bovis*. However, the active compounds identified from previous screens should display activity against *M*. *tuberculosis* to get cataloged as novel compounds having antitubercular potential. Thus, the MIC values for resultant 35 compounds identified from the current screen were determined against *H37Rv* in BSL3 facility. The obtained MIC values confirm that 28 compounds showed MIC ranging from >12.5 μm to 25 μm whereas 12 compounds showed MIC values in the range of 0.4 to12.5μm against *H37Rv* (Table 1). These 12 compounds were shortlisted to take them forward further analysis.

**Table 1:** Complete analysis of data from screening for antitubercular activity and physico chemical properties of the hit compounds. Rifampicin and Isoniazid were taken as controls. MIC values for controls against *M*. *smegmatis* are Rif-2.43±0.02μM and Inh-11.03±0.05 μM and *H37Rv* are Rif-0.08±0.01μM and Inh-0.22±0.3 μM.

S.No	Compound Name	*H37Rv* MIC (μM)	*M*. *smeg* MIC (μM)	BCG MIC (μM)	Bacteriocidal/Bacteriostatic	Molecular weight	ALogP	Hbond donors	Hbond Acceptors	PSA	No. of Rotatable Bonds
1	H15	12.5	41.99	12.02	Bacteriostatic	401	-1.478	1	6	67.6	5
2	H26	0.4	52.97	12.04	Bactericidal	368	1.156	1	5	72.19	3
3	H27	1.56	12.84	6.58	Bacteriostatic	393	0.995	1	6	95.98	3
4	H43	6.28	51.98	13.53	Bacteriostatic	366	-0.062	0	7	99.75	8
5	H85	12.5	25.13	11.51	Bacteriostatic	475	0.789	1	6	90.53	8
6	H94	6.25	25.36	12.70	Bacteriostatic	348	3.225	2	7	120.45	7
7	H117	12.5	26.79	14.90	Bacteriostatic	225	2.789	0	2	41.12	1
8	H126	12.5	30.61	23.83	Bacteriostatic	498	3.139	1	8	97.03	6
9	H127	12.5	46.56	14.20	Bacteriostatic	435	1.618	1	8	116.07	7
10	H134	12.5	22.70	10.88	Bacteriostatic	319	1.253	0	5	81.03	4
11	H136	1.6	42.48	11.39	Bactericidal	367	0.134	2	5	89.1	3
12	H137	12.5	39.20	22.75	Bacteriostatic	439	1.079	1	6	73.72	6

After the hits are identified in early discovery phase where compound libraries were screened in HTS mode needs to be reevaluated systematically towards lead generation. The lead compounds can undergo further optimization steps for lead optimization. First, we have evaluated chemical amenability of the 12 hits with the help of chemists to determine feasibility of re synthesis of these compounds in trouble-free chemical routes. Interestingly, the supervised analysis of hit compounds based on their chemical features revealed that they did not relate to each other very closely (data not shown). The MIC values of individual compounds also do not show any positive correlation may be due to diversity in chemical nature of the compounds identified. This fact also elated us that this observation circuitously proves that the library of compounds archived is diverse and not many similar compounds are present in any identified group. If they were to be present then they would have comparable activity in the biological screens. On the other hand because of the diverse nature, it is difficult to draw SAR as similarities are not easy to find. Out of the 12 HITs, two compounds were ureas (**H15** and **H94**) and three were pyrimidinones (**H137, H26** and **H27**). All the others were very diverse to develop any correlation. Probably they all act through different mode of action targeting diverse biological pathways for observed antituberculosis activity. This exercise has helped identify new scaffolds which can provide basis for developing novel drugs against tuberculosis.

Further, not to ignore the batch to batch variations with biological activity of the compounds the hit compounds were re synthesized using classical organic chemistry synthesis (Methodology for re-synthesis will be communicated elsewhere). The new batch of compounds was tested directly on *M*. *tb* strain *H37Rv* to determine their antimycobacterial potential. As presented in table 1 the compounds showed MIC values are comparable with the MIC determined in first screening. Taken together, the series of filters employed systematically to the data obtained from primary screening has led us in identifying new scaffolds with antimycobacterial properties and provides path for lead optimization phase for antitubercular drugs.

After identification of potential antituberculosis compounds from screening of large and chemically diverse libraries it is important to validate bactericidal activity of the best candidates to assess if selected molecules or scaffolds lead to develop new antimicrobial agents in the future. The MBC test allows determining whether the antimicrobial agents show bactericidal or bacteriostatic effect on growth of particular microorganism [20]. The MBC is the concentration of antimicrobial agent at which 99.99% of bacteria are killed by the test agents [39]. In order to better understand the potency of the compound, both MIC and MBC values are taken into consideration [21]. On the basis of comparison between MIC and MBC, compounds are categorized as either Bactericidal or Bacteriostatic. The definitions of “bacteriostatic” and “bactericidal” appear to be straightforward: “bacteriostatic” means that the agent prevents the growth of bacteria (arresting them in stationary phase of growth), and “bactericidal” means that it kills bacteria [40]. When the MIC and MBC values of a compound are equivalent the compound is considered as bacteriocidal [21]. A significant difference in MIC and MBC values of a compound suggests that the compound is bacteriostatic, wherein the MBC value is significantly higher than the MIC value [41].

In the present study, MBC test for the 12 hit compounds (Table 1) was performed as previously described against the test organisms chosen in the current study. From the assay results it is clear that compounds **H26** and **H136** showed MBC values equivalent to their MIC values where as other compounds have significantly higher MBC than their MIC values inhibiting bacterial growth. Hence, **H26** and **H136** display bactericidal activity against the tested microorganisms. As a result **H26** and **H136** showing bactericidal activity with potent MIC values (0.4 and 1.6 μM respectively) with good selectivity over cytotoxicity offer basis for chemistry to develop new antitubercular agents. It has been previously reported that when the MBC value is higher than the MIC value, the compound does not show bactericidal activity fairly acts as bacteriostatic arresting bacterial growth in stationary phase [42]. Antibiotics such as co-trimoxazole a sulfonamide antibiotic that showed MIC and MBC values as 31.2μg/ml and 125μg/ml respectively were categorized as bacteriostatic antibiotic [41]. Similarly, in the present study, 10 of the selected compounds showed similar differences in MBC and MIC values. Thus the observed MBC values for the remaining 10 compounds suggest that they bacteriostatic (Table 2). Currently many bacteriostatic inhibitors are in use to treat infections effectively [43]. Linezolid is an antibiotic categorized as bacteriostatic has been shown to demonstrate promising antitubercular activity [43,44]. Further, among antitubercular drugs such as ethambutol is bacteriostatic where as isoniazid and pyrazinamide are bactericidal to rapidly dividing bacteria and bacteriostatic to slowly dividing bacteria [45,46]. In the current study despite the fact that the hits with bacteriostatic potential showing MICs between 6.25 to 12.5 μM, they provide starting points to chemistry to develop these scaffolds further for new antitubercular agents.

**Table 2:** Cytotoxic evaluation of the hit compounds. Doxorubicin was taken as control. TI stands for therapeutic index calculated by IC_50_/GI_50_ value against *H37Rv*.

S.No	Compound Name	HEPG2		A549	
		IC_50_ (μM)	TI	IC_50_(μM)	TI
1	H15	509.96	>50	525.62	>50
2	H26	416.56	>50	367.99	>50
3	H27	444.23	>50	901.54	>50
4	H43	373.24	>50	334.55	>50
5	H85	790.08	>50	496.84	>50
6	H94	599.43	>50	443.31	>50
7	H117	407.81	>50	474.53	>50
8	H126	357.28	>50	369.45	>50
9	H127	347.5	>50	410.7	>50
10	H134	368.15	>50	423.85	>50
11	H136	359.12	>50	321.22	>50
12	H137	389.57	>50	385.09	>50
13	Doxorubicin	4.6	ND	5.2	ND

### Potential of identified scaffolds in tuberculosis drug discovery

**Fig 6:**
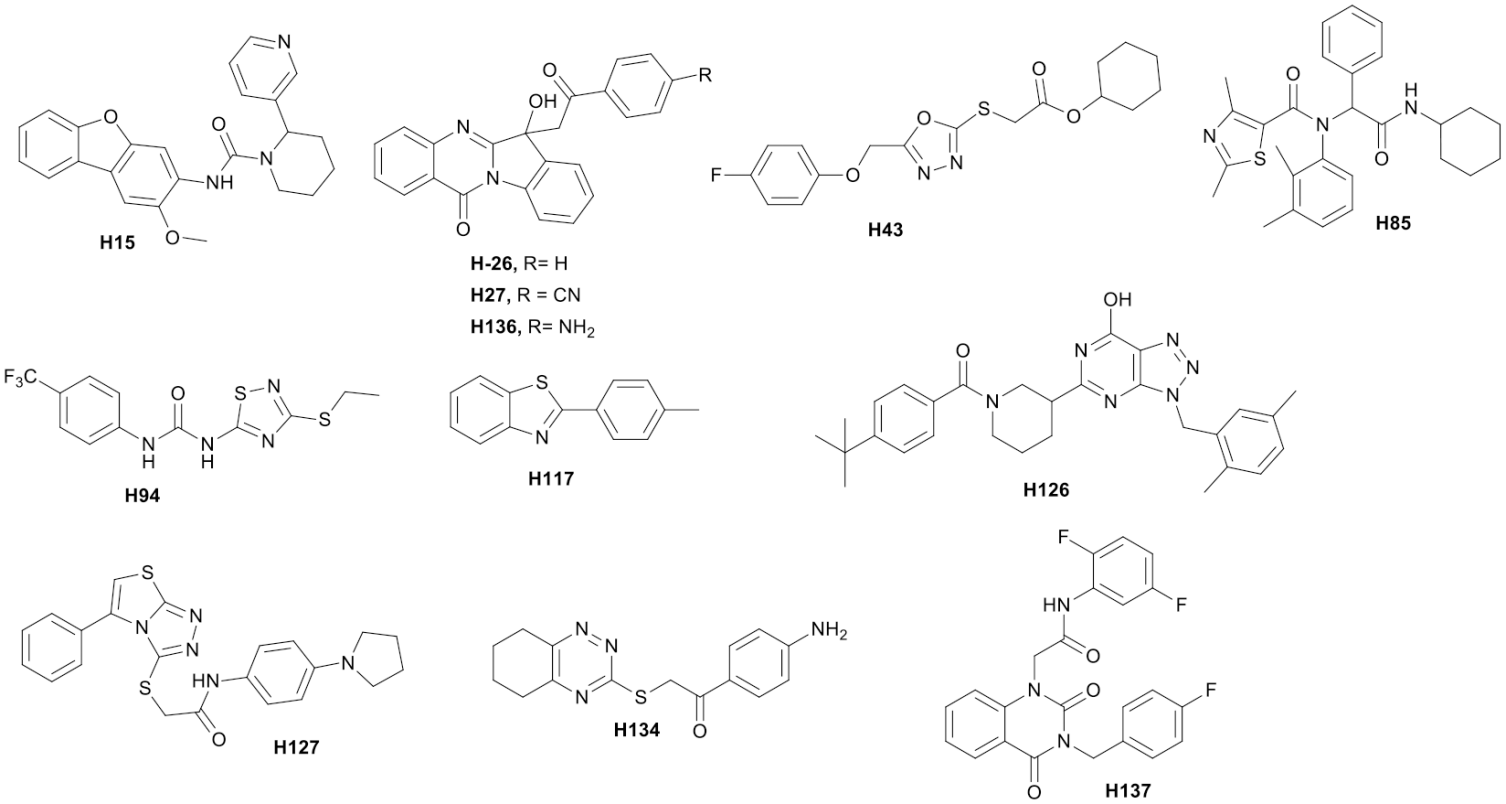
Potential scaffolds newly identified for the tuberculosis drug discovery.

The identified 12 chemically diverse hits can be classified in 10 scaffolds as shown in Fig 6. Hit **H15** is a 2-methoxydibenzofuran derivative and is chemically tractable providing ample scope for further chemical manipulations to identify more potent analogs. The tryptamine based hits **H26**, **H27** and **H136** are close analogues of Phaitanthrin A which was isolated from *Phailusmismensis* [47]. Also encouraging to note that the compound **H26** showed IC_50_ of more than 50 μM against MCF-7, NCI-H460, SF-264 describing its good selectivity index in killing bacteria [47]. In our hand, MIC for **H26** against *H37Rv* is 0.4 μM with less cytotoxicity against A549 and HepG2 cells (Table 2). Of note is that all the tryptanthrin analogs showed *M*. *tb* inhibition indicating the robustness of tryptanthrin scaffold as antitubercular agent and pave a way for further chemical modifications. The **H43** has oxadiazole scaffold while thiadiazole, its close scaffold, have been reported to display antifungal activities [48]. The **H85** is a thiazolecarboxamide derivative. **H94** is a substituted phenyl 1,2,4-thiadiazolyl-urea scaffold. Certain thiadiazoles have been reported to possess antibacterial activities against methiciline-resistant staphallycocusaures (MRSA) and *P*. *auregenosa* [49]. This background information supports the possibility to explore this scaffold further for the antitubercular activity. **H117** belongs to benzothiazole class and is a sole fragment in the identified hits. This fragment has potential for further exploration using fragment based drug discovery approach. **H126** has a triazolopyrimidin scaffold and has eight hydrogen bond acceptor and one hydrogen bond donor. The scaffold has more flexibility as it has five rotatable bonds. These physicochemical properties should allow designing better analogues of the series. **H127** is a fused thiazolo-triazol while **H134** has 1,2,4-benzotriazine scaffold. **H137** is a 1,3-disubstituted quinazoline-2,4(1H,3H)-dione scaffold and has not been explored previously for antitubercular activity. Few antituberculosis molecules are in various clinical trials. TBA-354 (nitroimidazole) is in phase I clinical trial whereas AZD5847 (oxazolidinone) and Sutezolid (oxazolidinone) are in the phase IIa clinical trial study. Delamanid (nitroimidazole), Pretomanid (nitroimidazole) and Bedaquiline (diarylquinoline) are in phase II clinical study. The identified scaffolds from our study are chemically different when compared with the above molecules/scaffolds which are at various clinical studies indicating the potential of these molecules for further modification for identification of new antituberculosis agents.

### Conclusion

In conclusion, from our phenotypic screening followed by filtering the hits based on properties for drug like molecules we have identified 103 compounds from a small molecule library made up of chemically diverse compounds. A total of 12 compounds have been identified based on the cutoff values MIC <12.5 μM against *H37Rv* and TI is >50. Further, confirmed hits are ranked based on the chemical diversity of the scaffolds and hit expansion around the identified scaffolds has been commenced in our lab. It is our hope that the hit expansion will generate novel compounds having a good drug likeness against *M*. *tuberculosis* and will enable us to develop novel leads to enter into preclinical drug development.

## Abbreviations

NMB: National MolBank
*M. tb*: *Mycobacterium tuberculosis*
NME: New Molecular Entities
